# Comparison of short-term clinical efficacy between percutaneous endoscopic transforaminal discectomy and unilateral biportal endoscopy in the treatment of upper lumbar disc herniation

**DOI:** 10.1186/s12891-026-09815-9

**Published:** 2026-04-15

**Authors:** Jing Zhang, Zhinan Ren, Lei Yu, Cheng Peng, Yingjie Hao

**Affiliations:** Department of Orthopaedics, The First Affiliated Hospital of Zhengzhou University, Zhengzhou, 450052 China

**Keywords:** Upper lumbar disc herniation, Percutaneous endoscopic transforaminal discectomy, Unilateral biportal endoscopy, Short-term clinical efficacy

## Abstract

**Objective:**

To compare the short-term clinical efficacy of percutaneous endoscopic transforaminal discectomy (PETD) with that of unilateral biportal endoscopy (UBE) in the treatment of upper lumbar disc herniation (ULDH).

**Methods:**

Clinical data from 109 consecutive hospitalized patients who were diagnosed with ULDH and admitted to the Department of Orthopaedics, The First Affiliated Hospital of Zhengzhou University, from February 2021 to February 2024 were retrospectively analysed. The patients were divided into two groups on the basis of the surgical approach: the PETD group (*n* = 58) and the UBE group (*n* = 51). Perioperative and follow-up indicators, including operation time, intraoperative blood loss, postoperative bed rest duration, postoperative hospital stay, postoperative complication rates, Oswestry Disability Index (ODI) scores, and Visual Analogue Scale (VAS) scores for low back and leg pain (assessed preoperatively and at 3 days, 3 months, and 6 months postoperatively), were compared. Additionally, the recurrence rate and excellent/good efficacy rate (evaluated via the modified MacNab criteria) were recorded at 12 months postoperatively. For imaging evaluation, lumbar MRI was used to assess changes in the dural sac cross-sectional area (DSCA) at the operated segment preoperatively and 6 months postoperatively.

**Results:**

No statistically significant differences were observed between the two groups in terms of operation time or intraoperative blood loss (*P* > 0.05). In both groups, the VAS scores for low back and leg pain and the ODI scores at 3 days, 3 months, and 6 months after surgery were significantly lower than those before surgery (*P* < 0.05); however, the intergroup differences in these scores at each time point were not statistically significant (*P* > 0.05). There were also no significant differences between the two groups in terms of the 12-month postoperative excellent/good efficacy rate or recurrence rate (*P* > 0.05). Regarding imaging results, both groups showed a significant increase in DSCA compared to preoperative values (*P* < 0.05), and no significant difference was found between the PETD and UBE groups (*P* > 0.05). Notably, compared with the UBE group, the PETD group had a shorter postoperative bed rest duration and hospital stay (*P* < 0.05). The overall incidence of postoperative complications was significantly lower in the PETD group than in the UBE group (5.17% vs. 21.17%, *P* < 0.05). In particular, the incidence of nerve injury (especially transient nerve injury) was also significantly lower in the PETD group (*P* < 0.05).

**Conclusion:**

Both PETD and UBE are effective for the short-term treatment of ULDH. Nevertheless, PETD offers advantages, including a lower incidence of postoperative complications (especially transient nerve injury), minimal intraoperative trauma, earlier postoperative ambulation, and a reduced duration of postoperative bed rest and hospital stay.

## Introduction

Upper lumbar disc herniation (ULDH) is defined as disc herniation occurring at the T12–L1, L1–2 or L2–3 levels. However, there is no international consensus on whether L3–4 disc herniation should be included in ULDH, and discrepancies in relevant case reports further reflect this lack of uniformity [1–5]. Sanderson et al. reported that the anatomical characteristics and surgical outcomes of L3–4 disc herniation did not differ significantly from those at lower lumbar levels. In contrast, postoperative improvements in low back pain and radicular pain were significantly poorer in patients with L1–2 or L2–3 herniation than in those with L3–4 herniation [6, 7]. Therefore, L3–4 disc herniation was not classified as ULDH in the present study. Clinically, ULDH is far less prevalent than lower lumbar disc herniation, accounting for only approximately 1%–11% of all cases of lumbar disc herniation [8, 9]. Anatomically, the upper lumbar spine is in close proximity to the conus medullaris. Unlike lower lumbar disc herniation, which typically compresses individual nerve roots, herniation at the upper lumbar levels often affects the dural sac, resulting in multifocal nerve compression within the sac [10]. This anatomical distinction contributes to the atypical clinical manifestations of ULDH: patients commonly present with pain and numbness in the anterior thigh, lateral thigh, or inguinal region, accompanied by hip and knee weakness. These symptoms differ substantially from those of the classic sciatica associated with lower lumbar pathology, rendering ULDH prone to misdiagnosis as a hip or knee joint disorder or genitourinary system disease [11–14].

At present, most scholars advocate surgical intervention as the first-line treatment for confirmed ULDH, with the primary goal of maximizing decompression of intraspinal neural structures. Spontaneous remission of ULDH is rare, and conventional conservative therapies (bed rest, physical therapy, and pharmacotherapy) generally yield suboptimal outcomes [15]. Surgical options for ULDH are broadly classified into open surgery and minimally invasive endoscopic spinal surgery. Traditional open surgery, however, is associated with a high risk of severe complications, including extensive iatrogenic trauma, increased intraoperative blood loss, and widespread dissection of paraspinal muscles and ligaments. These factors not only cause significant damage to posterior spinal soft tissues but also predispose patients to late-stage complications such as secondary lumbar instability and intractable low back pain. Consequently, minimally invasive endoscopic spinal techniques have gained increasing interest among clinicians and patients alike [16, 17].

Despite the widespread adoption of minimally invasive spinal surgery for the treatment of lumbar disc herniation, a comprehensive review of current literature revealed that most comparative studies of percutaneous endoscopic transforaminal discectomy (PETD) and unilateral biportal endoscopy (UBE) focus on lower lumbar disc herniation, hence leaving a gap in direct comparative evidence of their efficacy, safety, and technical feasibility in ULDH. Two retrospective cohort studies had evaluated the individual application of PETD and UBE in the treatment of ULDH; although they reported promising short-term outcomes, the relatively small sample size limited the statistical power of the analyses [8, 18]. One systematic review and meta-analysis of minimally invasive techniques for ULDH revealed that there is no evidence supporting the superiority of any single minimally invasive approach. This research gap indicates that clinicians lack objective data to guide selection of the most appropriate minimally invasive technique for ULDH patients. Current clinical practice is largely based on surgeon experience rather than evidence-based guidelines, creating an urgent need for comparative studies to guide clinical decision-making [3, 19]. Therefore, the present study was designed to investigate and compare the short-term clinical efficacy of PETD with that of UBE in the treatment of ULDH.

## Materials and methods

### Patients

Clinical data from 109 consecutive hospitalized patients who were diagnosed with ULDH and underwent either PETD or UBE in the Department of Orthopedics, The First Affiliated Hospital of Zhengzhou University, from February 2021 to February 2024 were analysed retrospectively. Based on the calculations for estimated clinical outcome measures, the required sample size was determined to be no less than 86 cases using PASS software. This ensures adequate power to detect clinically significant differences in the outcomes evaluated. The surgical approach for each patient was ultimately determined through multidisciplinary discussion between the surgical team and radiologists to avoid individual preference bias. This study was approved by the Ethics Committee of The First Affiliated Hospital of Zhengzhou University (approval number: 2025-KY-1698). All the participants signed an informed consent statement. All surgical procedures were performed by attending surgeons from the same surgical team with equivalent professional qualifications and at least 5 years of clinical experience in spinal endoscopic techniques. Each surgeon was proficient in both PETD and UBE techniques, having completed over 80 cases of each procedure, thereby ensuring a consistent level of surgical proficiency between the two groups.

### Inclusion and exclusion criteria

#### Inclusion criteria

(1) A preoperative diagnosis of single-segment ULDH (T12–L1, L1–2 or L2–3) confirmed by a combination of clinical symptoms and imaging examinations, including digital radiography (DR), computed tomography (CT), and magnetic resonance imaging (MRI); (2) symptoms that failed to improve or worsened after ≥ 3 months of standardized conservative treatment (medication, physical therapy, and back muscle functional exercise); (3) ≥ 12 months of outpatient follow-up, with complete clinical and imaging records.

#### Exclusion criteria

(1) A history of spinal surgery at the responsible segment; (2) radiological evidence of significant instability at the responsible segment, as indicated by dynamic lumbar X-rays (intervertebral body translation > 3 mm or angular motion > 15° between adjacent vertebrae) [20, 21]; (3) severe pathological changes at the responsible segment, including massive disc calcification (occupying > 50% of the disc space) [22] or extensive posterior vertebral osteophyte hyperplasia; (4) concurrent spinal diseases at the responsible segment, such as spinal tumours, lumbar pyogenic infection or lumbar tuberculosis; (5) multi-segment lumbar disc herniation (herniation involving two or more lumbar segments, including both upper and lower lumbar levels).

### Surgical procedures

#### PETD group

**Fig. 1:**
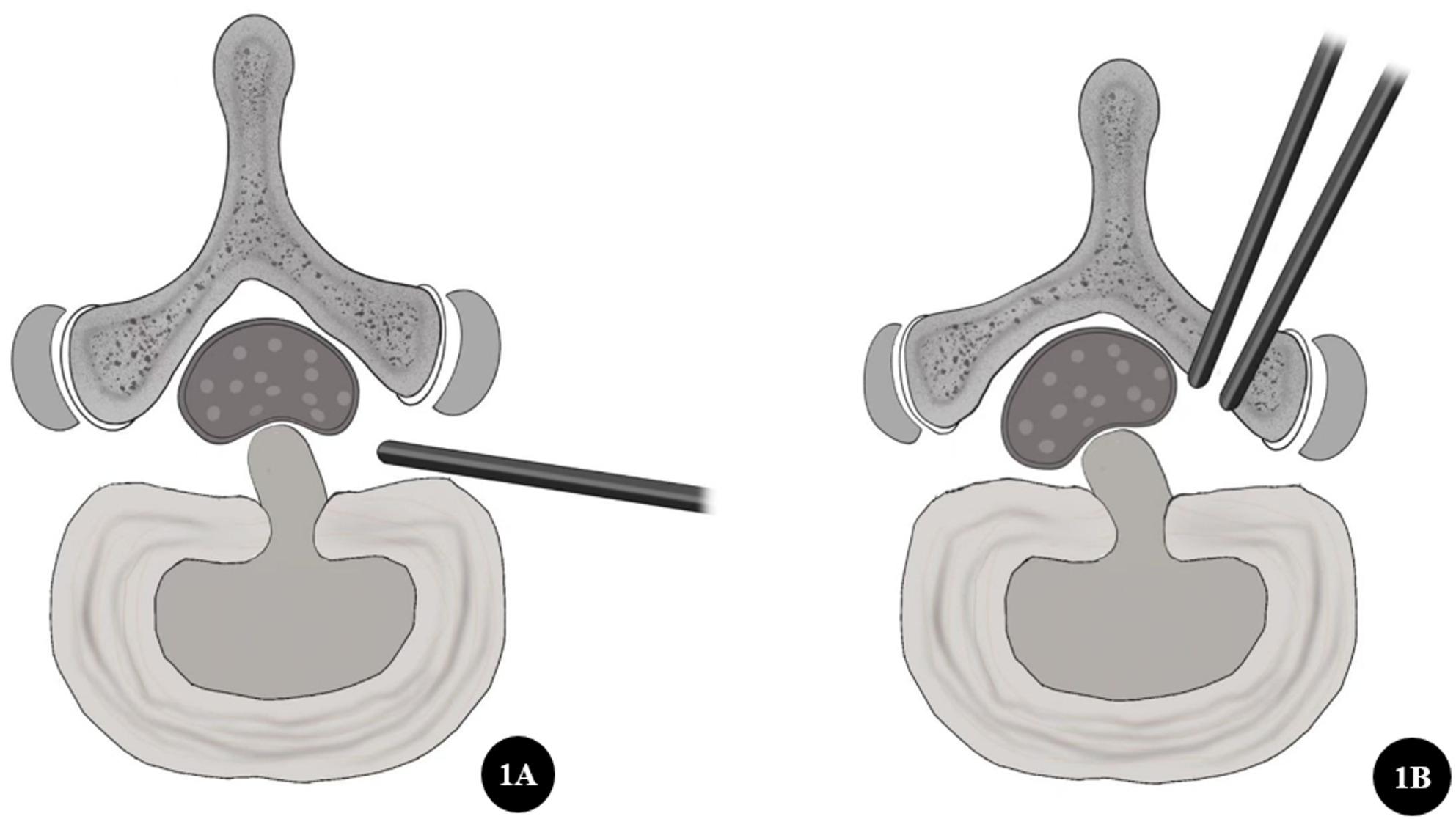
Schematic Diagram of PETD (**A**) and UBE (**B**)

The patient was positioned prone with bilateral chest and abdominal padding to maintain abdominal suspension. The intervertebral foramen of the responsible segment was located using C-arm fluoroscopy. Following standard disinfection and draping, a diluted solution of lidocaine and ropivacaine was used for local infiltration anaesthesia, which extended to the zygapophyseal joint capsule. The puncture needle was inserted step by step, and its optimal position was confirmed via anteroposterior and lateral C-arm fluoroscopy. The needle core was then removed, and a guide wire was inserted. The skin and soft tissues were dilated sequentially using tapered dilators, and then a working cannula was placed along the guide wire. The position of the working cannula was reconfirmed under fluoroscopy before the intervertebral foramen endoscope was inserted. Under endoscopic visualization, a portion of the hypertrophic ligamentum flavum was resected using nucleus pulposus forceps to access the spinal canal and expose the herniated nucleus pulposus. The sequestered or protruded nucleus pulposus was meticulously removed with nucleus pulposus forceps. Intraoperative re-evaluation confirmed adequate decompression, as evidenced by brisk dural pulsation, relaxed nerve roots, and the absence of residual nucleus pulposus in the spinal canal. Annuloplasty was subsequently performed using radiofrequency ablation. The working sleeve and endoscope are removed, and the incision was closed. See Fig. 1 for details.

#### UBE group

Following endotracheal intubation under general anaesthesia, the patient was positioned prone with bilateral chest and abdominal padding to maintain abdominal suspension. The cutaneous projection of the pedicle at the responsible segment was located using C-arm fluoroscopy. After standard disinfection and draping, two parallel 1-cm incisions were made at the pedicle projection on the ipsilateral side of the herniation: one for the observation channel and the other for the working channel. The skin and soft tissues were incised layer by layer. Blunt dissection of the paraspinal muscles was performed using a periosteal elevator to create a tissue plane. A high-speed burr was used to resect a portion of the inferior articular process of the upper vertebra and the superior articular process of the lower vertebra to expand the surgical corridor. Under endoscopic visualization, the herniated nucleus pulposus was meticulously removed with nucleus pulposus forceps. Intraoperative re-evaluation confirmed adequate decompression, as evidenced by brisk dural pulsation, relaxed nerve roots, and the absence of residual nucleus pulposus in the spinal canal. Annuloplasty was subsequently performed using radiofrequency ablation. The working sleeve and endoscope were removed, and the incision was closed. See Fig. 1 for details.

#### Postoperative management

Both groups received standardized postoperative care, including intravenous administration of nonsteroidal anti-inflammatory drugs for analgesia, and prophylactic antibiotics for 24 h. Patients in the PETD group were permitted to ambulate with a lumbar corset under surgeons’ supervision on postoperative day 1. In contrast, those in the UBE group initiated ambulation with the same lumbar corset only after the wound drainage tube was removed (a daily drainage volume < 50 mL) [23]. The surgical site was dressed every 2–3 days. Dressing changes were continued for a total of 2 weeks until complete epithelialization of the incision was confirmed. All patients were scheduled for outpatient follow-up at 3, 6, and 12 months postoperatively. During the 3-month postoperative period, patients were instructed to avoid frequent forward bending, heavy lifting, and strenuous physical labor. They were also instructed to perform appropriate lumbar and back muscle function exercises.

#### Observation indicators

Primary outcomes: (1) ODI scores and VAS scores were measured preoperatively and at 3 days, 3 months, and 6 months postoperatively to evaluate changes in lumbar function and pain intensity. (2) Efficacy of modified MacNab and recurrence were evaluated at 12 months postoperatively. Postoperative recurrence was defined as follows: after initial endoscopic discectomy for lumbar disc herniation, patients achieved symptom relief for ≥ 6 months, followed by recurrent lower extremity pain, sensory disturbance, muscle weakness or other neurological dysfunction. All recurrent cases were confirmed on MRI, which showed reherniation of residual nucleus pulposus at the surgical level, with a distinct disc fragment identified on the ipsilateral or contralateral side [24, 25]. (3) Lumbar MRI scans were obtained preoperatively and at 6 months postoperatively to measure the dural sac cross-sectional area (DSCA) on T2-weighted axial images at the operative level. DSCA was assessed by two independent spine surgeons who were blinded to the group allocation, and the final result was taken as the mean of their respective measurements. DSCA improvement rate = (postoperative DSCA - preoperative DSCA)/preoperative DSCA × 100%.

Secondary outcomes: Perioperative data, including operation time, intraoperative blood loss, postoperative bed rest duration, postoperative hospital stay and postoperative complications, were collected for both groups. Complications were categorized as cerebrospinal fluid (CSF) leakage, nerve injury, epidural hematoma, wound infection, and lower extremity deep vein thrombosis (DVT). Postoperative lumbar CSF leakage was diagnosed on the basis of the following criteria: ① intraoperative confirmation of dural sac injury with CSF leakage, accompanied by postoperative drainage of clear fluid or massive light-red bloody fluid; ② postoperative development of intracranial hypotension symptoms (postural headache, nausea, vomiting); ③ postoperative sustained increase in light-red or clear fluid drainage from the incision tube within 24 h; ④ persistent exudation of light-red or clear fluid from the surgical incision; ⑤ aspiration of light-red or clear fluid from subcutaneous effusion via puncture; ⑥ radiological evidence of CSF leakage on imaging [26, 27]. Postoperative neurological status was assessed through a combination of patient self-report and clinical physical examination by the attending surgeons, electromyography examination should be performed when necessary [28].

### Statistical analysis

Data were analyzed using SPSS software (Version 23.0, Chicago, IL, USA) and PASS15.0 software. Continuous variables were expressed as mean ± standard deviation and categorical variables were expressed as quantity or proportion. All continuous variables were confirmed to follow a normal distribution by the Kolmogorov–Smirnov test, and homogeneity of variance was assessed by Levene’s test. Intergroup comparisons of continuous variables were employed using independent sample t-test and intragroup comparisons were conducted using paired sample t-test. Categorical variables were compared using chi-square test and Fisher’s exact test, depending on the expected frequencies. One-way repeated measures ANOVA was used to analyze intragroup scores at different time points. The effect size of the primary outcome was calculated. Comparisons with values of *P* < 0.05.

## Results

### General information

**Fig. 2:**
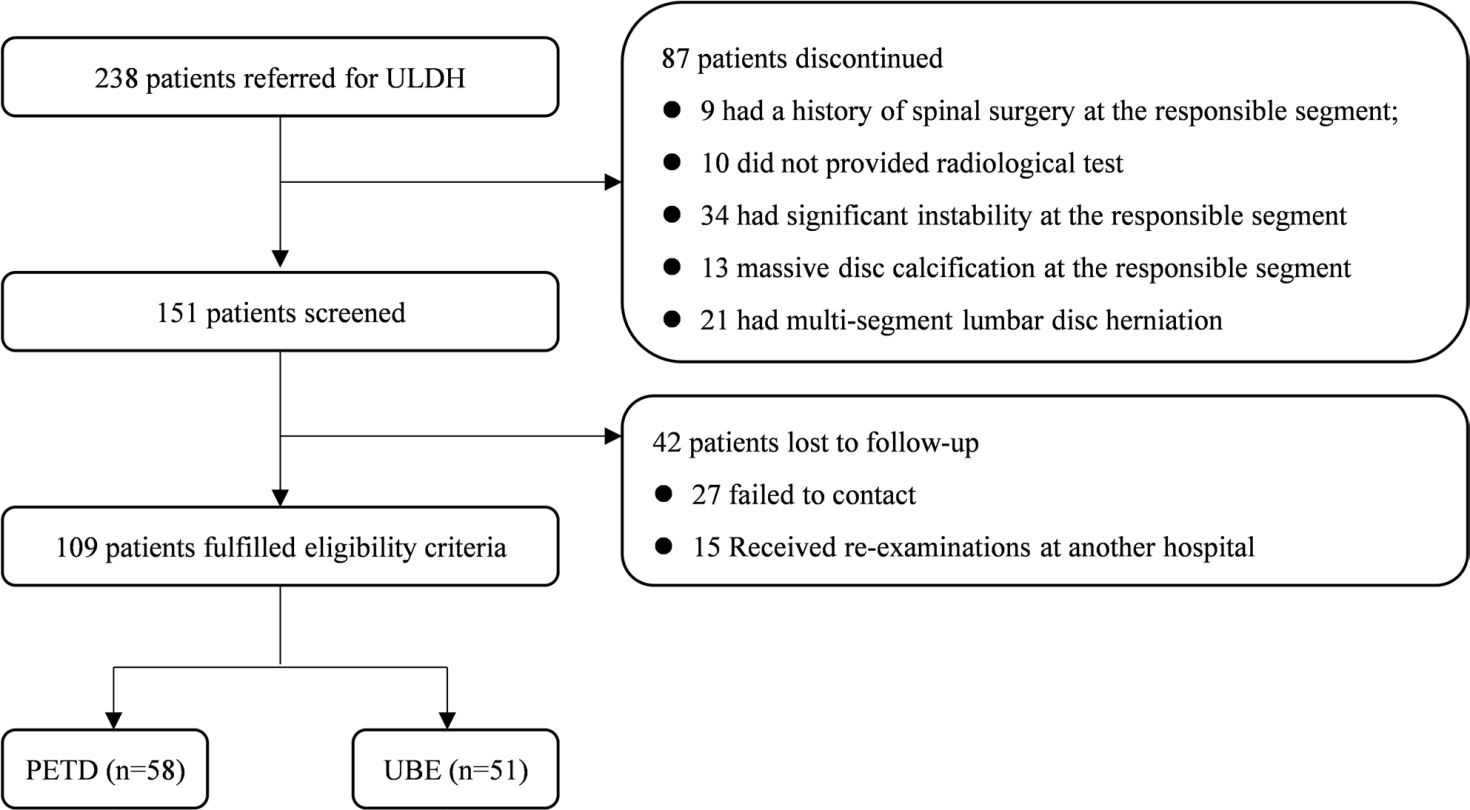
The flowchart of the study

**Fig. 3:**
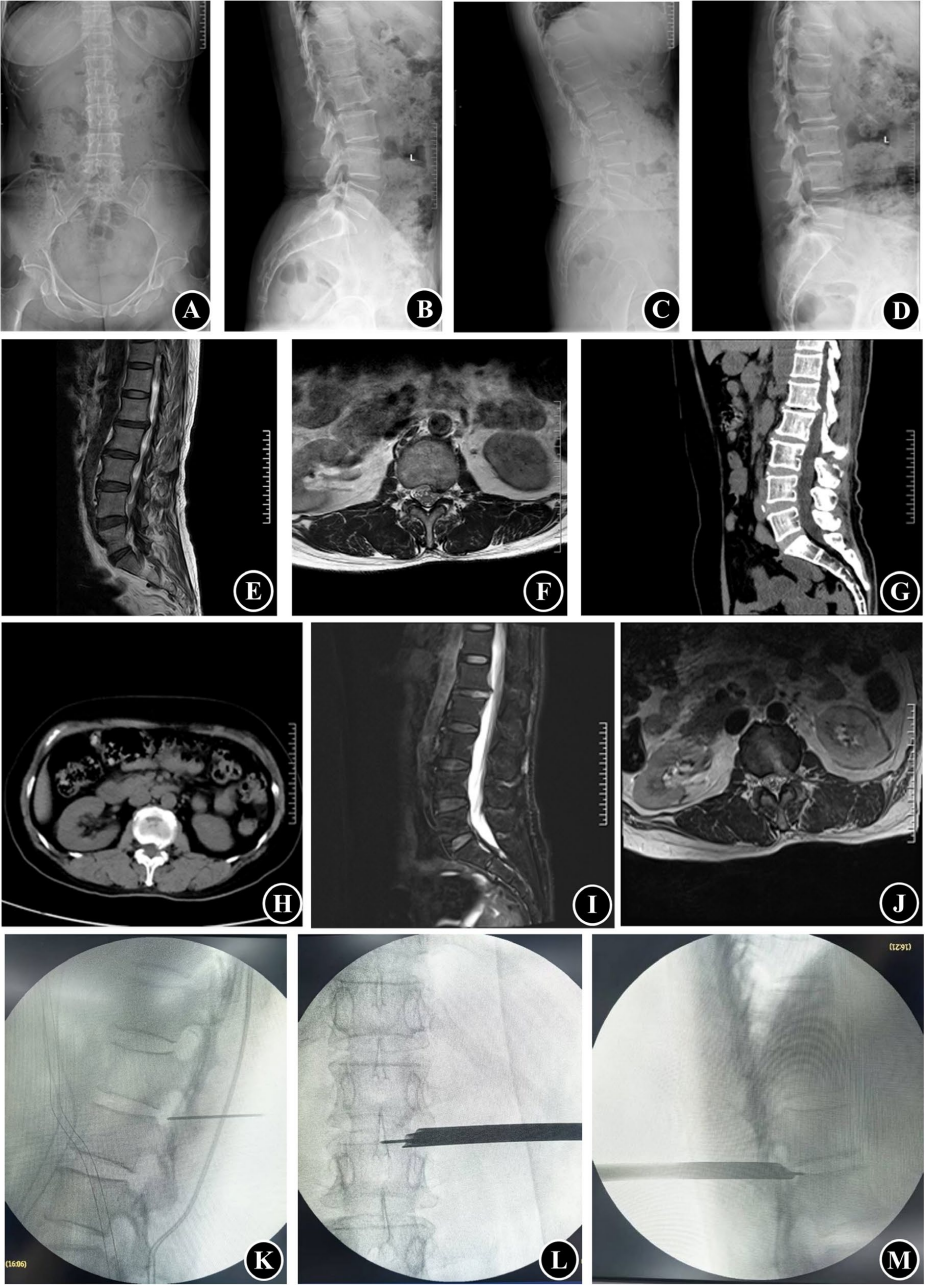
PETD group: A 45-year-old female presented with a 15-month history of low back pain accompanied by left anterior thigh pain. **A–D**: Preoperative lumbar X-rays, mild lumbar degenerative changes and scoliosis, with no radiological evidence of significant lumbar instability; **E–F**: Preoperative lumbar MRI, left-sided disc herniation at the L1/2 level; **G–H**: Preoperative lumbar CT, mild calcification of the left-sided herniated disc at the L1/2 level; **I–J**: Postoperative lumbar MRI reexamination, complete resolution of the L1/2 left-sided disc herniation and adequate decompression of the previously compressed L2 nerve root; **K–M**: Intraoperative C-arm fluoroscopy localization

**Fig. 4:**
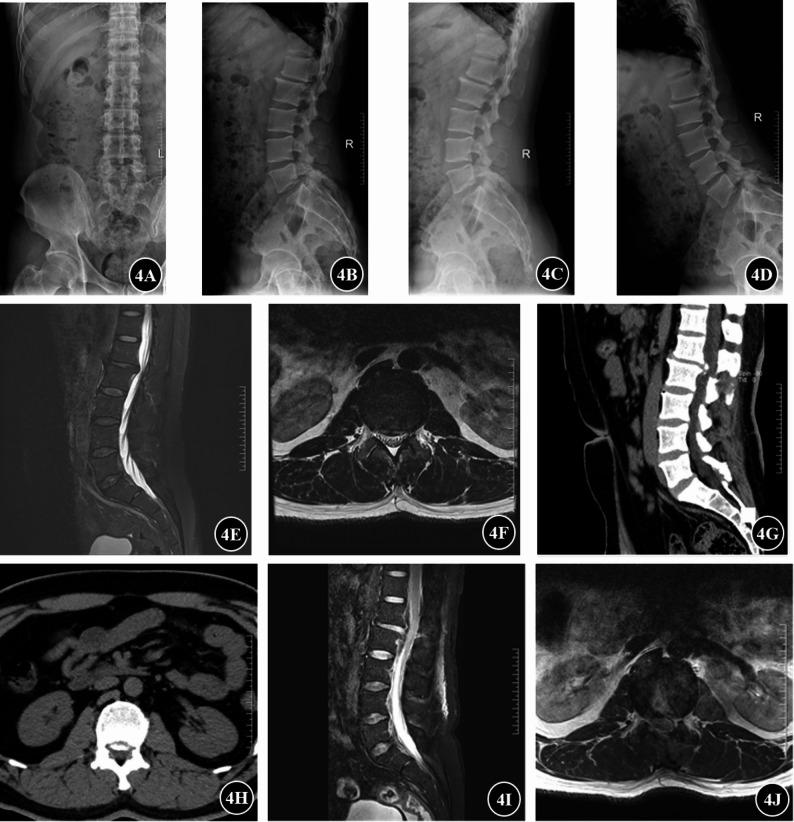
UBE group: A 33-year-old male presented with a 1-year history of low back pain and a 6-month history of bilateral thigh weakness (left side more severe). **A–D**: Preoperative lumbar X-rays, straightening of the lumbar physiological lordosis, along with limited range of motion in hyperextension and hyperflexion; **E–F**: Preoperative lumbar MRI, central disc herniation at the L1/2 level, with significant compression of the dural sac and bilateral L2 nerve roots; **G–H**: Preoperative lumbar CT, central herniation of the L1/2 disc with focal calcification; **I–J**: Postoperative lumbar MRI reexamination, Sagittal view: Complete removal of the herniated nucleus pulposus and restoration of dural sac morphology; Axial view: Partial lamina defect on the left surgical side and adequate bilateral neural decompression

In the PETD group (*n* = 58), there were 32 males and 26 females; the age range was 18–50 years, with a mean age of 34.26 ± 4.81 years; and the disease duration ranged from 6 to 20 months, with a mean duration of 13.95 ± 2.48 months. In the UBE group (*n* = 51), there were 28 males and 23 females; the age range was 18–60 years, with a mean age of 35.92 ± 5.57 years; and the disease duration ranged from 10.45 to 20.53 months, with a mean duration of 14.46 ± 2.16 months. No statistically significant differences were observed in baseline characteristics—including sex, age, body mass index (BMI), disease duration, type of herniation, and involved spinal segment—between the two groups (*P* > 0.05; Table 1). Continuous variables (age, BMI, disease duration) were compared using the independent sample t-test; Categorical variables (sex, type of herniation, involved spinal segment) were compared using the chi-square test. Figure 2 shows the flowchart of the study. All 109 patients completed surgery uneventfully. Representative cases (one from each group) are illustrated in Figs. 3 and 4.

**Table 1:** Comparison of general information between the two Groups

	PETD	UBE	t/χ2	*P*	Effect size
Sex			0.010	0.920^*^	0.010
male	32	28			
female	26	23			
Age(years)	34.26 ± 4.81	35.92 ± 5.57	1.657	0.101^*Δ*^	0.321
BMI(Kg/m^2^)	22.68 ± 3.24	23.15 ± 2.96	0.791	0.431^*Δ*^	0.151
disease duration(months)	13.95 ± 2.48	14.46 ± 2.16	1.149	0.253^*Δ*^	0.218
Surgical segment			0.048	0.976^*^	0.015
T12–L1	10	8			
L1–2	18	16			
L2–3	30	27			
Type of herniation			0.093	0.954^*^	0.021
Central	12	10			
Paracentral	35	32			
Foraminal / Extraforaminal	11	9			

Δ indicates independent sample t-test; ^*^indicates chi-square test

### VAS scores for low back and leg pain and ODI scores

**Fig. 5:**
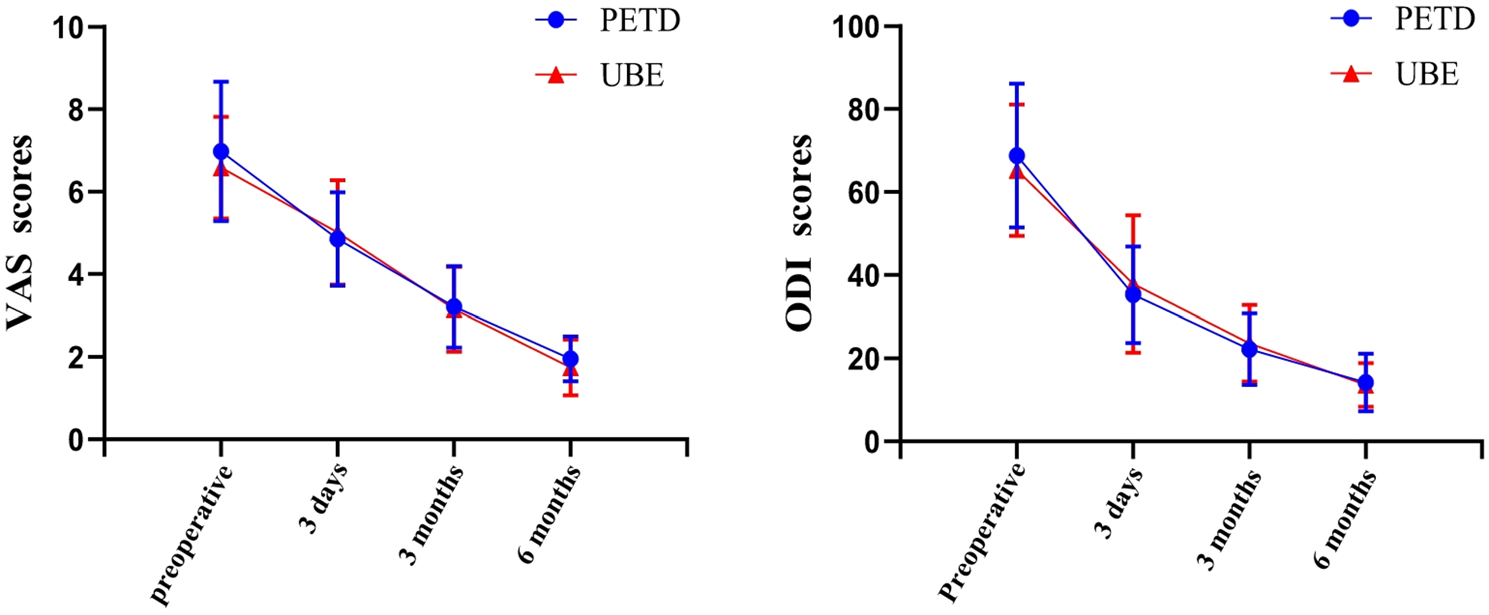
Line chart of preoperative and postoperative changes in VAS and ODI scores

For both the PETD and UBE groups, the VAS scores for low back and leg pain and the ODI scores at 3 days, 3 months and 6 months postoperatively were significantly lower than the preoperative values, and the difference was statistically significant (*P* < 0.05). However, the results of the intergroup comparisons revealed no statistically significant differences in the VAS scores and the ODI scores at any postoperative time point (*P* > 0.05). See Table 2 and Fig. 5 for details.

**Table 2:** VAS scores for low back and leg pain and ODI scores

	PETD	95% CI	UBE	95% CI	t	*P*	Effect size
VAS scores							
Preoperative	6.98 ± 1.69	(6.53, 7.43)	6.59 ± 1.23	(6.24, 6.94)	1.388	0.168^*Δ*^	0.267
3 days after surgery	4.85 ± 1.13	(4.56, 5.16)	5.02 ± 1.27	(4.66, 5.38)	0.734	0.465^*Δ*^	0.141
3 months after surgery	3.21 ± 0.98	(2.96, 3.48)	3.15 ± 1.02	(2.86, 3.44)	0.312	0.755^*Δ*^	0.060
6 months after surgery	1.95 ± 0.54	(1.81, 2.09)	1.74 ± 0.68	(1.54, 1.90)	1.769	0.080^*Δ*^	0.330
*F*	214.360		192.850				
*P*	< 0.001^✝^		< 0.001^✝^				
ODI scores							
Preoperative	68.82 ± 17.38	(64.76, 74.28)	65.29 ± 15.82	(61.28, 70.08)	1.110	0.269^*Δ*^	0.213
3 days after surgery	35.29 ± 11.62	(32.51, 38.79)	37.91 ± 16.52	(32.72, 41.80)	0.945	0.348^*Δ*^	0.182
3 months after surgery	22.17 ± 8.59	(20.95, 25.51)	23.62 ± 9.18	(21.75, 26.93)	0.848	0.398^*Δ*^	0.163
6 months after surgery	14.24 ± 6.91	(12.43, 16.19)	13.59 ± 5.27	(12.00, 15.18)	0.556	0.579^*Δ*^	0.107
*F*	209.747		187.623				
*P*	< 0.001^✝^		< 0.001^✝^				

^✝^indicates one-way repeated measures ANOVA for multiple time points; ^Δ^indicates independent sample t-test

### Efficacy of modified MacNab and recurrence rate

At the 12-month postoperative follow-up, the rate of excellent/good efficacy, according to the modified MacNab criteria, did not significantly differ between the PETD group and the UBE group (*P* > 0.05). A total of 4 cases recurred in the PETD group and 3 cases in the UBE group, the postoperative recurrence rate at 12 months was not significantly different between the two groups (*P* > 0.05). All recurrent patients initially received conservative treatment, including analgesics, neurotrophic drugs, physical therapy and restricted weight-bearing activities. Among them, only 1 patient in the UBE group underwent revision surgery after conservative treatment failed, and the remaining patients in both groups achieved satisfactory symptom relief with conservative treatment. See Table 3 for details.

**Table 3:** Efficacy of modified MacNab and recurrence rate^**^

Modified MacNab criteria	PETD	UBE	*P*	Effect size	*P*	OR(95%CI)
Excellent	52	42				
Good	4	6				
Fair	1	2				
Poor	1	1				
Excellent + Good	96.55%(56/58)	94.12%(48/51)	0.686^*#*^	0.068	0.674^**^	0.646 (0.084, 4.94)
Postoperative recurrence rate	6.89%(4/58)	5.88%(3/51)	1.000^*#*^	0.021		

^#^indicates Fisher’s exact test; ^**^indicates ordinal logistic regression analysis

### Comparison of imaging parameters between the two groups

Preoperatively, there was no significant difference in DSCA on axial lumbar MRI between the two groups (*P* > 0.05). At 6 months postoperatively, intragroup comparisons showed that DSCA was significantly increased in both groups compared with preoperative values (*P* < 0.05). Intergroup comparisons demonstrated no significant difference in DSCA (*P* > 0.05). See Table 4 and for details. Statistical analysis was performed to assess the correlations, and no significant correlations were identified between the DSCA improvement rate and the reduction rates of VAS and ODI scores (*P* > 0.05). This result indicates that postoperative clinical improvement may not be determined by the extent of radiological decompression.

**Table 4:** Comparison of imaging parameters between the two groups

	PETD	UBE	t	*P*	Effect size
DSCA (mm^2^)					
Preoperative	73.86 ± 7.49	75.09 ± 7.61	0.850	0.397^*Δ*^	0.163
6 months after surgery	122.54 ± 8.62	123.55 ± 8.95	0.598	0.550^*Δ*^	0.115
DSCA improvement rate (%)	67.40 ± 19.16	66.33 ± 21.94	0.270	0.788^*Δ*^	0.052
*t*	54.89	41.39			
*P*	< 0.001^***^	< 0.001^***^			

^Δ^indicates independent sample t-test; ^*^indicates paired t-test within the same group; ^***^indicates paired t-test within the same group

### Comparison of perioperative indicators between the two groups

There were no statistically significant differences in operation time or intraoperative blood loss between the two groups (*P* > 0.05). Notably, compared with the UBE group, the PETD group had a significantly shorter duration of postoperative bed rest and hospital stay (*P* < 0.05). Continuous variables (operation time, intraoperative blood loss, postoperative bed rest duration) were compared using the independent sample t-test.

The overall incidence of postoperative complications was significantly lower in the PETD group (5.17%) than in the UBE group (21.57%). The incidence of nerve injury in the PETD group (especially transient nerve injury) was significantly lower than that in the UBE group (*P* < 0.05). See Table 5 for details. Transient nerve injury was defined as postoperative neurological symptoms (sensory disturbance, mild muscle strength weakness, or radicular pain) with a duration of < 3 months. These symptoms resolved completely after conservative treatment and resulted in no permanent neurological dysfunction. Categorical variables (postoperative complications) were compared using the chi-square test. In the PETD group, a patient with transient nerve injury presented with exacerbation of lower extremity pain. Symptomatic treatment with neurotrophic and blood circulation-promoting drugs was administered, and the pain resolved before discharge. In the UBE group, 5 patients had transient nerve injury: 3 patients had aggravated lower extremity pain, and 2 patients had lower extremity sensory disturbance, their symptoms were completely resolved at the 3-month postoperative follow-up; 1 patient developed persistent urinary dysfunction: follow-up at 12 months showed no improvement, and urodynamic testing confirmed bladder detrusor contraction dysfunction; and 1 patient had lower extremity muscle strength decline: preoperative muscle strength was grade 4, which decreased to grade 3 at 3 days postoperatively; at the 6-month follow-up, muscle strength recovered to grade 4– (slightly below the preoperative level), and full recovery (grade 5) was achieved by 12 months postoperatively. No statistically significant differences were observed between the two groups in terms of the incidence of other complications, including CSF leakage, epidural hematoma, wound infection and DVT (*P* > 0.05). See Table 6 for details.

**Table 5:** Comparison of perioperative data between the two groups

	PETD	UBE	t/χ2	*P*	Effect size
Operation time (min)	135.91 ± 25.42	128.69 ± 21.37	1.611	0.109^Δ^	0.309
Intraoperative blood loss (ml)	57.62 ± 13.36	61.25 ± 14.97	1.344	0.181^*Δ*^	0.258
Postoperative bed rest duration (h)	12.35 ± 2.06	23.18 ± 7.21	10.361	< 0.001^*Δ*^	2.102
Postoperative hospital stay (d)	4.28 ± 0.85	5.36 ± 0.93	6.334	< 0.001^*Δ*^	1.212
Postoperative complication					
Nerve injury	1	7	NA	0.042^*#*^	0.511
CSF leakage	1	2	NA	0.611^*#*^	0.085
Epidural hematoma	0	2	NA	0.248^*#*^	0.136
Wound infection	1	0	NA	1.000^*#*^	0.092
DVT Incidence of complication	0 5.17%(3/58)	0 21.57%(11/51)	NA 6.458	NA 0.011^*^	NA 0.508

^Δ^indicates independent sample t-test; ^*^indicates chi-square test; ^#^indicates Fisher’s exact test

**Table 6:** Postoperative neurological complications and outcomes

	Type	Cases	Clinical Manifestation	Outcomes
PETD	Transient nerve injury	1	Exacerbation of lower extremity pain	Symptom relief
UBE	Transient nerve injury	5	3 cases: aggravated lower extremity pain 2 cases: lower extremity sensory disturbance	Symptom relief
	Urinary dysfunction	1	Urinary retention	No improvement
	Muscle strength decline	1	Pre-op: Grade 4 Post-op day 3: Grade 3	Full recovery (grade 5)

## Discussion

ULDH is a relatively rare clinical disorder, so high-quality clinical evidence comparing the efficacy of PETD and UBE for this condition remains scarce. In our study, the significant improvements in pain score and functional status observed in both groups at 3 days, 3 months and 6 months postoperatively were consistent with prior findings. Additionally, the modified MacNab criteria revealed an acceptable patient satisfaction and imaging results showed adequate postoperative dural sac decompression in UBE and PETD groups, indicating that both techniques were equally effective in the treatment of ULDH. However, PETD technique demonstrated distinct advantages, including a significantly shorter postoperative bed rest duration, postoperative hospital stay and a lower complication rate, particularly a reduced incidence of nerve injury.

The superior perioperative outcomes of PETD observed in this study can be primarily attributed to four key factors. (1) The upper lumbar intervertebral foramen has a larger diameter than that in the lower lumbar spine, which permits direct access to and resection of the herniated nucleus pulposus via the transforaminal approach during PETD without the need for bone removal. In contrast, UBE adheres to the principles of open surgery and requires partial resection of the zygapophyseal joints and lamina to create an adequate working corridor [29, 30]. This bony resection can subtly impair segmental lumbar stability, often necessitating prolonged bed rest to mitigate the risk of early instability-related complications. (2) PETD is performed under local anesthesia, with the patient remaining awake throughout the procedure. This enables direct communication between the surgeon and patient, so any intraoperative neural irritation can be reported immediately. Such feedback allows for the timely adjustment of surgical maneuvers, thereby minimizing the risk of iatrogenic nerve injury [31, 32]. However, UBE requires general anesthesia and surgeons must rely entirely on anatomical familiarity and intraoperative tactile feedback to evaluate nerve tension. This reliance increases the risk of unrecognized over-traction or compression of neural structures [33]. (3) In PETD, the surgeon accesses the herniated nucleus pulposus via the intervertebral foramen, thereby avoiding direct manipulation of intraspinal neural elements such as the conus medullaris and cauda equina. This approach minimizes mechanical nerve stimulation and reduces the risk of injury. In contrast, UBE utilizes an interlaminar approach to enter the spinal canal, necessitating dissection and operation in close proximity to neural bundles, which inherently elevates the potential for nerve irritation or trauma [34]. (4) Both techniques rely on continuous normal saline irrigation to maintain a clear surgical field, yet they differ in their pressure requirements. UBE necessitates higher irrigation pressure to expand the paraspinal soft tissue and create an adequate working cavity, which permits non-interfering movement of the endoscope and instruments. Excessive pressure, however, risks impairing neurological function [35, 36]. PETD, by contrast, is performed in a naturally formed intervertebral foramen space and requires lower irrigation pressure.

Xu et al. reported comparable complication rates between PETD and UBE for lower lumbar disc herniation [37]. In contrast, our study demonstrated that the UBE group had a significantly higher incidence of nerve injury at the upper lumbar spinal segments. This difference stems from the unique anatomical and biomechanical characteristics of the upper lumbar spine. Anatomically, the upper lumbar spinal canal is mostly oval-shaped, while the lower lumbar spinal canal is triangular or clover- shaped. Notably, the cross-sectional area of the upper lumbar spinal canal is relatively small [38]. Moreover, the conus medullaris and dense cauda equina nerves pass through the upper lumbar spinal canal, where the distribution of epidural fat is scarce, resulting in a limited buffer space for the nerves. In contrast, the lower lumbar spinal canal mainly contains the cauda equina nerves, with abundant cerebrospinal fluid between nerve bundles providing effective cushioning [38, 39]. Therefore, the unique anatomical characteristics of the upper lumbar spine present distinct surgical challenges for ULDH: the narrower operative space increases technical difficulty, and even minor intraoperative traction or manipulation risks irreversible neurological complications, including bilateral lower extremity paraplegia or neurogenic bladder dysfunction. In contrast, neurological injury during surgery for lower lumbar disc herniation generally affects only a single nerve root’s innervated area and does not result in paraplegia of both lower extremities [40].

PETD is a percutaneous single-channel endoscopy procedure through the Kambin’s triangle, which demands precise puncture, fluoroscopic identification, and accurate in vivo endoscopic orientation. Its working space is relatively confined, leading to a steeper learning curve. Surgeons require rigorous training and sufficient case volume to master the core techniques of accurate puncture and endoscopic decompression [41]. By contrast, UBE establishes separate viewing and working channels through two small incisions, providing a wide surgical field comparable to open surgery. Therefore, it has a gentler learning curve and is more readily mastered by novice surgeons. Previous studies revealed that the cutoff point required to overcome the learning curve of PETD surgery was 40 cases, while for UBE, it was 15 cases [42]. The dual-channel design of the UBE provides a broader surgical field and greater freedom of instrument manipulation, which makes it more effective in addressing complex spinal lesions such as massive intraspinal calcification and enables contralateral decompression without excessive bony resection [43]. However, owing to its transforaminal approach, PETD is more suitable for patients with foraminal or extraforaminal lumbar disc herniation, while demonstrating limited ability in treating severely calcified lesions. In clinical practice, the surgical strategy should be individualized [44]. Key considerations include patient-specific factors and the surgeon’s proficiency with each technique. A thorough preoperative evaluation and in-depth patient–physician communication are essential to ensure that the chosen approach aligns with the patient’s condition and treatment goals.

This study had several limitations. Firstly, the relatively small sample size and short-term follow-up limited the statistical efficacy of the analyses and may have impaired the identification of uncommon complications. Secondly, as a retrospective analysis, it is subject to inherent limitations: the non-randomized allocation of patients and the reliance on retrospectively collected clinical data introduce the potential for selection bias. Finally, this study was performed in a single-center, which may limit the generalizability and external validity of the findings. In future studies, we plan to conduct multi-center prospective randomized controlled trials (RCTs) with a larger sample size and a longer follow-up period of more than 2 years to further evaluate the long-term efficacy, safety, and generalizability of these minimally invasive techniques.

.

## Conclusion

Our study indicates that both PETD and UBE are effective for the short-term treatment of ULDH. PETD offers advantages, including lower incidences of postoperative complications (especially transient nerve injury), minimal intraoperative trauma, earlier postoperative ambulation, and a reduced duration of postoperative bed rest and hospital stay.

## Data Availability

The datasets used and analysed during the current study are available from the corresponding author on reasonable request.
